# Paratesticular Angiomyofibroblastoma-Like Tumor: Unusual Case of a Solidocystic Form

**DOI:** 10.1155/2017/1273531

**Published:** 2017-02-28

**Authors:** Issam Msakni, Dorra Ghachem, Mohamed Amine Bani, Faten Gargouri, Nada Mansouri, Ramzi Khiari, Fethi Bougrine, Ammar Bouziani, Besma Laabidi

**Affiliations:** ^1^Department of Pathology, Military Hospital, Tunis, Tunisia; ^2^Department of Urology, Military Hospital, Tunis, Tunisia

## Abstract

The angiomyofibroblastoma-like tumor of the male genital tract is a rare benign tumor. A total of 34 cases have been reported in the literature. We herein report an exceptional case of solidocystic form in its paratesticular location, in a 79-year-old man. Clinical examination objectified a right testicular induration. Macroscopic examination of the orchidectomy objectified a paratesticular solidocystic tumor formation. Microscopically, the solid area was composed of vessels with small caliber and turgidity of endothelial cells. These vessels were surrounded by clusters of epithelioid cells, sometimes having the appearance of giant cells. They were associated with spindle cells. The cystic area was uncoated. Immunohistochemically, the fusiform cells expressed Desmin and Smooth Muscle Actin. Endothelial cells and clusters of cells surrounding large vessels expressed CD34. The expression of receptors for estrogen and progesterone was negative. To our knowledge, this is the second solidocystic case of angiomyofibroblastoma-like tumor of male genital tract.

## 1. Introduction

The angiomyofibroblastoma-like tumor of the male genital tract is a rare benign tumor [1, 2]. Its appellation is based on the similarities with cellular angiofibroma of the female genital tract [3]. The histogenesis of this tumor involves conjunctive perivascular cells [1–3]. Thirty-three cases have been reported describing a solid mass [2]. We report an exceptional case of solidocystic form of paratesticular angiomyofibroblastoma-like tumor.

## 2. Case Presentation

**Figure 1:**
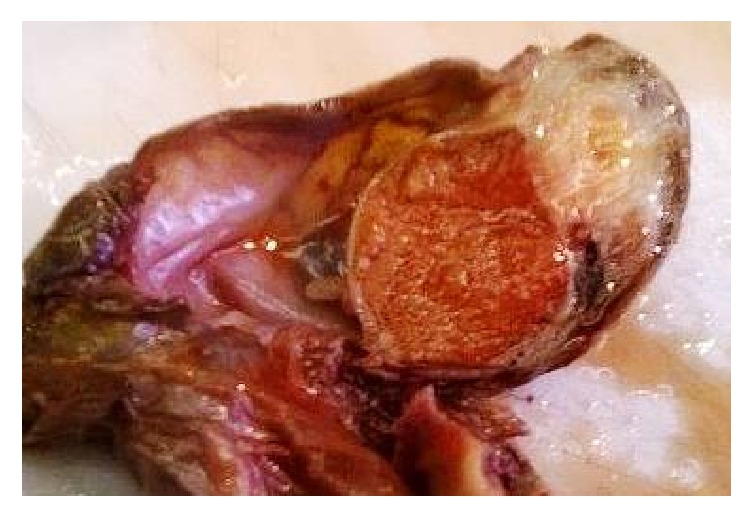
Well-limited solidocystic paratesticular tumor.

**Figure 2:**
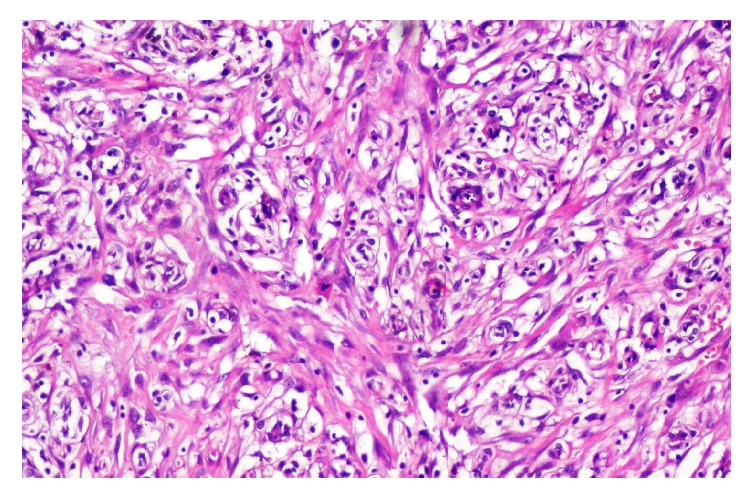
Proliferation of small caliber blood vessels with turgescent endothelial cells, surrounded by clusters of epithelioid cells, and associated with spindle cells proliferation.

**Figure 3:**
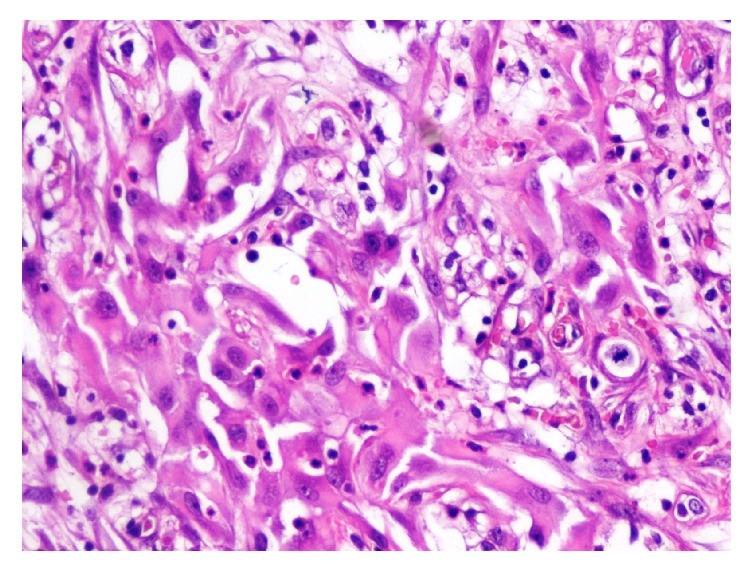
Epithelioid cells have large and big regular nuclei. See the presence of mitosis with no atypia.

**Figure 4:**
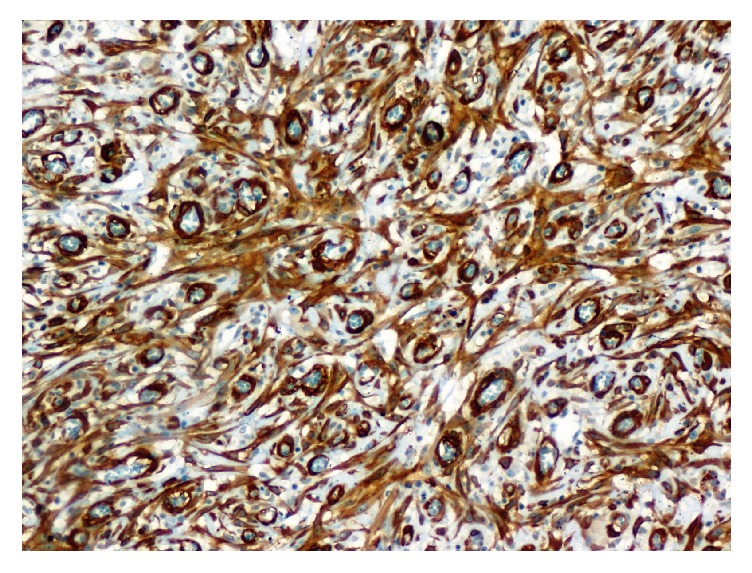
Smooth Muscle Actin immunostaining ×200. Diffuse and intense staining of the spindle cells surrounding the vessels.

**Figure 5:**
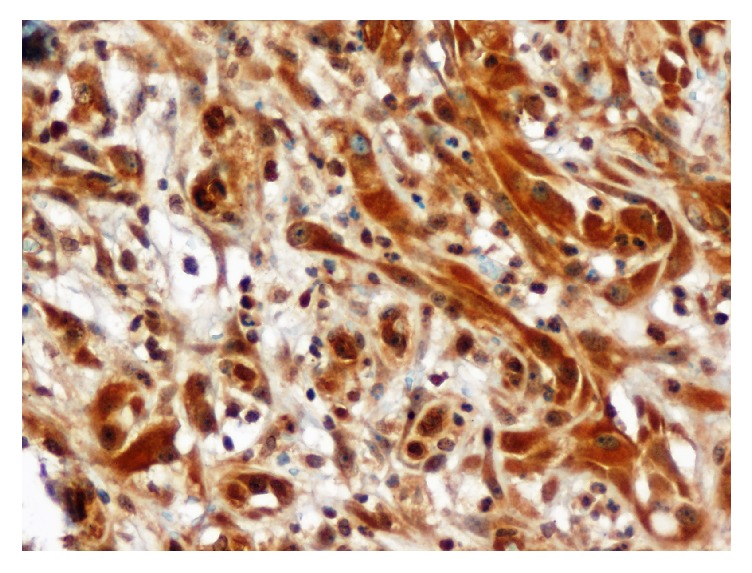
CD34 immunostaining ×400. An intense expression of CD34 by the endothelial cells and the epithelioid cells.

A 79-year-old man, with no medical history, consulted for burning in urination and urinary leakage. Clinical examination revealed a right testicular induration. Normal levels of lactic dehydrogenase (LDH), alpha-fetal protein, and human chorionic gonadotropin (hCG) were found. Ultrasonography showed a paratesticular hypoechogenic mass measuring 3.4 cm. It was located at the right epididymal tail and encompassing the adjacent testicular parenchyma. A right orchidectomy was performed. The specimen measured 9 × 5 × 3 cm. It was composed of a solidocystic tumor. The cyst measured 5 cm in its great diameter with a yellowish liquid content. This cyst was in continuity with a solid area measuring 2 × 1 cm above an atrophic testicle measuring 3 × 3 × 1.5 cm. The spermatic cord was unremarkable measuring 11 × 2 cm (Figure 1). The histological examination showed a well-limited tumor of small blood vessels. The endothelial cells were turgescent, enlarged in size, with abundant cytoplasm. The nuclei were regularly big and clarified. There was no atypia. These vessels were surrounded by clusters of epithelioid cells, large with big regular nuclei. Some of these cells had an appearance of giant cells. They were mixed with spindle cells around the vessel walls (Figures 2 and 3). The cyst wall was made of a loose fibrous tissue without any epithelium. On immunohistochemistry, the spindle cells were intensely and diffusely staining for the Desmin and Smooth Muscle Actin (AML) (Figure 4). The large epithelioid cells and the endothelial cells were intensely positive for CD34 and CD31 (Figure 5). The progesterone and estrogen receptors were negative. Given the histological findings, as well as the immunohistochemistry, the final diagnosis was a cystic angiofibroblastoma-like tumor.

## 3. Discussion

The angiofibroblastoma-like tumor is a rare tumor with only 34 observations described in the literature [1, 2]. This tumor is benign, well-circumscribed, whose size varies between 2.5 cm and 14 cm with an average of 6.7 cm [2–4]. In the present case, the size of the tumor was 7 cm (the solid area measured 2 cm and the cystic area 5 cm). It is usually asymptomatic but may sometimes be painful [3]. The majority of reported cases occur at an advanced age as in the present case (between 50 and 80) [3]. The most commonly affected sites are the inguinal scrotal and vulvovaginal regions [1]. In our case, the location is paratesticular. This tumor was described in 1997 as a cellular angiofibroma. Its name was changed to angiofibroblastoma-like tumor by Laskin et al. in 1998; similarities were observed with angiofibroblastoma described tumor in women in 1992. The testicular tumor markers, which are the hCG and alpha-fetoprotein, are at a normal rate as that described in our case [5]. Testicular ultrasound usually shows a solid hypoechoic lesion [1, 3]. Macroscopically, the tumor is usually solid. One case of solidocystic form was reported by de Souza et al. in a 19-year-old young man. The location was at the inguinal canal [6]. To our knowledge, this case is the second reported case of solidocystic form of this tumor. Bifocal angiofibroblastoma-like tumor had been described by Hsu et al. [1]. Histologically, the nomenclature, angiofibroblastoma, is based on the two main components of the tumor: blood vessels and connective tissue. This has been observed in the majority of reported cases [1–9]. The tumor is well circumscribed, even encapsulated [1, 2, 5, 7, 10]. Intralesional fat tissue can sometimes be present [1–3]. A lipomatous variant has been described [8, 10]. In this case, the mature adipocytes occupy more than 50% of tumor volume [8]. Atypia, mitosis, and necrosis are usually absent [2–4, 7, 9, 11]. Rare mitoses (between 1 and 4 mitoses/50 high power fields) were observed in other reported cases [4, 5, 7, 8]. Scattered cytonuclear atypia were reported by João et al. [3]. In more than half of the reported cases, the tumor cells are positive for CD34 and are variable with Desmin and AML. They may express focally estrogen or progesterone receptors, whereas they are negative to protein S-100 [4, 9]. In this case, the immunohistochemical study showed positivity for CD34, CD31, Desmin, and AML. Negativity to estrogen and progesterone receptors was found. The histogenesis of the tumor is most likely due to a myofibroblastic differentiation from a stem cell under the influence of cytokines and growth factors, which expresses the CD34 antigen [5, 7]. The study of Iwasa and Fletcher examined 51 cases of cellular angiofibroma in inguinoscrotal localization of 25 men and vulvovaginal localization of 26 women. CD34 was expressed in 60% of the cases, SMA in 21% of the cases, and Desmin in 8% of the cases; S-100 protein was negative in all the cases [2]. The main differential diagnosis is the angiomyxoma which also develops in the pelvic and perineal region. This tumor is characterized by its aggressive behaviour as it is locally infiltrative and invasive without metastatic potential. Recurrences are frequent. On immunohistochemistry both angiomyofibroblastoma-like tumor and aggressive angiomyxoma are positive for Desmin and SMA. The best criterion to differentiate between them is the high cellularity and the more pronounced vasculature in the angiomyofibroblastoma-like tumor, while in aggressive angiomyxoma, smooth muscle cells are characteristically around the vessels as in the angiomyolipoma [7]. The myxoid interstitial also directs the diagnosis towards angiomyxoma. Tumor regression has been seen in angiomyofibroblastoma [8]. Only one case of tumor recurrence after 13 years has been reported, and no cases of sarcoma or pejorative evolution transformation have yet been described [1, 4, 5, 8]. The recommended treatment is the surgical excision with clear wide margins. A postoperative follow-up on the short term and long term is necessary [5, 6, 11].

## 4. Conclusion

Angiofibroblastoma-like tumor is a rare tumor and information regarding its characteristics is lacking. Clinical presentation has no pathognomonic signs. The solidocystic variant has only been reported in one case and its histological and immunohistochemical profiles are characteristic. This report provides an update of the most current findings and adds to the available data concerning this tumor.
